# Fluorescent Photoelectric Detection of Peroxide Explosives Based on a Time Series Similarity Measurement Method

**DOI:** 10.3390/s23198264

**Published:** 2023-10-06

**Authors:** Weize Shi, Yabin Wang

**Affiliations:** School of Mechatronical Engineering, Beijing Institute of Technology, Beijing 100081, China; shiweize@bit.edu.cn

**Keywords:** peroxide explosives, fluorescent photoelectric detection, time series, DDTW, Spearman correlation coefficient

## Abstract

Due to the characteristics of peroxide explosives, which are difficult to detect via conventional detection methods and have high explosive power, a fluorescent photoelectric detection system based on fluorescence detection technology was designed in this study to achieve the high-sensitivity detection of trace peroxide explosives in practical applications. Through actual measurement experiments and numerical simulation methods, the derivative dynamic time warping (DDTW) algorithm and the Spearman correlation coefficient were used to calculate the DDTW–Spearman distance to achieve time series correlation measurements. The detection sensitivity of triacetone triperoxide (TATP) and H_2_O_2_ was studied, and the detection of organic substances of acetone, acetylene, ethanol, ethyl acetate, and petroleum ether was carried out. The stability and specific detection ability of the fluorescent photoelectric detection system were determined. The research results showed that the fluorescence photoelectric detection system can effectively identify the detection data of TATP, H_2_O_2_, acetone, acetonitrile, ethanol, ethyl acetate, and petroleum ether. The detection limit of 0.01 mg/mL of TATP and 0.0046 mg/mL of H_2_O_2_ was less than 10 ppb. The time series similarity measurement method improves the analytical capabilities of fluorescence photoelectric detection technology.

## 1. Introduction

Peroxide explosives represent a new type of explosive containing peroxy bonds, represented by TATP. Its molecular formula is C_6_H_18_O_6_, and its melting point is 94 °C. TATP is insoluble in water and soluble in organic solvents at room temperature. It can be sublimated and volatilized. TATP has the characteristics of easily available materials, simple production, and high explosive power. It is very sensitive to impact, friction, static electricity, high temperature, etc., and has low thermal stability. Therefore, the detection method for it is also different from traditional explosive detection methods. The detection methods for peroxide explosives such as TATP mainly include infrared spectroscopy, Raman spectroscopy, electrochemistry, ion mobility spectrometry, mass spectrometry, chemical colorimetry, liquid chromatography, fluorescence detection, etc.

Both infrared absorption spectroscopy and Raman scattering spectroscopy record molecular spectra based on molecular vibration and rotation [1,2]. Different vibration and rotation forms can cause changes in the dipole moment and polarizability of a molecule. TATP is a typical small organic molecule and thus has characteristic Raman and infrared spectral responses. Van used near-infrared (NIR) spectroscopy technology to conduct the spectral characterization of explosives such as 2,4,6-trinitrotoluene (TNT), cyclotrimethylene trinitramine (RDX), pentaerythritol tetranitrate (PETN), and TATP, which can prevent false positive results caused by household chemicals, explosive raw materials, and drugs of abuse [3]. However, inorganic materials such as black powder still cannot be effectively identified. Spencer determined the peak of the Raman spectral fingerprint of TATP powder through Raman spectral analysis to achieve the detection of TATP [4]. Wackerbarth designed a surface-enhanced Raman spectroscopy sensor for the on-site detection of TATP gas, which selectively adsorbed TATP molecules and could achieve the sensitive detection of TATP gas within 30 s [5]. Based on a green laser pointer, Malka developed a modular and compact Raman spectrometer with a detection limit of 1 ng [6]. However, due to the very weak Raman and infrared characteristic spectra, the spectral database and spectral recognition algorithm still require further research. Arman used the electrochemical detection method and polyethyleneimine to modify a glassy carbon electrode to achieve the direct detection of TATP and hexamethylene triperoxide diamine (HMTD) without the catalytic decomposition of H_2_O_2_ and obtained a detection limit of 1.5 mg/L TATP [7]. Krivitsky used silver nanoparticles to modify carbon microfiber electrodes without interference from other organic compounds, and the detection limit for gaseous TATP reached 10^−5^ mg/L in laboratory tests [8]. However, the detection time of the electrochemical method was long, and the detection could not be performed in a portable manner. Li designed an ultra-fast polarity-switching ion mobility spectroscopy (IMS) drift tube with a non-radioactive ionization source, and employed novel methods in the electronic design to switch the aperture grid and ion shutter, with the detection limit of 0.1 ng [9]. Mamo reported an electrospray ionization ion mobility spectrometer with both positive and negative ion modes capable of detecting explosives such as ammonium nitrate (AN), TNT, and TATP [10]. However, the cost was higher, and the detection time was longer. Parajuli used an electrochemical sensor to detect TATP in the liquid phase. TATP could also be detected in the presence of other types of explosives, such as PETN and RDX, and the laboratory detection limit could reach the μg/L level [11]. Hilton used the electrochemiluminescence method to quantitatively detect the intensity of light generated via the electrochemical reaction of TATP, and the laboratory detection limit reached 2.5 μmol [12]. However, the anti-interference ability was poor, and the method was easily affected by various environmental impurities. Sağlam used PBE-memory polycarbazole films decorated with gold nanoparticles (AuNPs) via cyclic voltammetry and designed an electrochemical sensor that can directly detect TATP and HMTD, with a detection limit of 15 μg/L [13]. Pintabona used an ionization mass spectrometry method in a surface acoustic atomization environment and detected seven kinds of nitro organic explosives (in the negative ionization mode), including TNT, and the two peroxide explosives (in the positive ionization mode) TATP and HMTD [14]. Hormozi reported a TATP chemical colorimetric method based on MnO_2_ nanozymes using acetic acid to catalyze decomposition to produce H_2_O_2_, which could be oxidized to change the sample color from colorless to brown, with a detection limit of 0.34 mg/L of TATP [15]. However, it took 30 min for there to be a response with the method, and the waiting time was long. Schulte used ultraviolet light to photochemically decompose TATP to obtain H_2_O_2_, catalyzed the reaction via catalase to generate fluorescent polymers, and quantitatively determined TATP explosives via liquid chromatography [16,17,18]. Based on the ambient ionization mass spectrometry method, Burns used an atmospheric-pressure solids analysis probe (ASAP) and secondary electrospray ionization (SESI) technology to analyze five common explosives including HMTD, RDX and TNT, with the detection limit of 0.8–10 ng [19]. However, this method is relatively expensive and still technically challenging.

Fluorescence detection is based on changes in photophysical properties such as the peak position of fluorescence emission and the intensity of the fluorescence caused by energy or electron transfer between fluorescent probe molecules and analyte molecules, and detection is performed by analyzing the response. Sugandha reported cyanostilbene-boronate probe 4, based on aggregation-induced emission enhancement (AIEE), which showed enhanced fluorescence emission in HEPES buffer, with good selectivity and sensitivity, and the calculated detection limit was 15.51 ppb [20]. Zhang reported a fluorescent thin-film probe for H_2_O_2_ vapor detection using a fluorene–pyrene compound linked with two boronate units, with a quenching rate of 62% within 120 s and a detection limit of 2.2 ppb for H_2_O_2_ vapor [21]. Yu reported a perylene diimide nanofiber–solid acid blend as an enhanced fluorescent probe for the detection of trace TATP vapor, with a detection limit of 0.1 ppm in a laboratory environment, and the actual detection time was 5 s [22]. Gökdere [23] and Fan [24,25] used the ion exchange resin Amberlyst-15 (hereinafter referred to as A-15) to catalyze the decomposition of TATP to generate H_2_O_2_ to activate the fluorescence of boronate. Zhu prepared portable fluorescent test paper made of curcumin derivatives, which was sensitive to H_2_O_2_ solution [26]. They also carried out simulation calculations for TATP, which showed a good detection effect, but the actual detection effect for TATP was not experimentally studied. Vargas synthesized a carboxyphenyl porphine dye with high-efficiency fluorescence properties, which could be oxidized by TATP molecules to change the conjugated structure of the dye, resulting in a fluorescence-quenching effect, and prepared a fluorescent sensor for the direct detection of TATP [27]. However, the sensor required more than 10 min for the reaction time.

In summary, in previous studies, infrared spectroscopy and Raman spectroscopy provided fingerprint spectra and accurate identification; electrochemical detection, ion mobility spectrometry detection and chemical colorimetric detection had high detection sensitivities and low detection limits; and mass spectrometry detection and identification were accurate. At present, the above methods have realized the detection of trace amounts of TATP, but due to technical limitations, they cannot meet the actual needs in practical applications. Take common explosives detection technology as an example. Although Raman spectroscopy technology has detection specificity, the equipment cost is high, and the Raman spectrum is extremely weak, meaning it requires strict detection conditions. IMS technology is mature, but has a high detection limit and long detection time, among other problems. Colorimetric detection is convenient and fast, but has large errors and poor trace detection results. Mass spectrometry (MS) technology is extremely costly and is only suitable for laboratory environmental sample analysis, and cannot be used in security inspection, logistics and practical applications in other fields. Fluorescence detection has the advantages of high sensitivity, a low detection limit, and a short detection time. Research has realized the detection of trace amounts of TATP, and high-sensitivity fluorescence detection has shown good results. However, related papers mainly focus on the research of fluorescent detection materials. There are problems such as inaccurate numerical simulation calculations and high detection limits in actual measurement experiments.

Recently, the time series similarity measurement method has been widely used in aerospace, finance, medicine, energy and other fields for pattern matching, data mining and classification. For example, based on the normalized difference vegetation index (NDVI) time series derived from the moderate resolution imaging spectroradiometer (MODIS), Guan used the DTW algorithm to calculate the difference between the NDVI time series of each pixel in the MODIS image and the standard rice growth NDVI time series [28]. Similarly, the results indicate that this method can be used to determine large-area rice cultivation systems with flexible rice cultivation schedules. In addition, the time series similarity measurement method has also been applied in research such as the t0ime series analysis of satellite attitude variation processes, cluster analysis of securities’ index time series, and short-term cardiovascular time series analysis. These studies are very similar to those of explosive fluorescence detection technology, and they are all data analyses related to time series. In this study, we processed collected weak fluorescence signals into stable voltage signals to obtain analyzable time series data that change over time. Compared with the time series with a long time and sparse data points in the above study, the time series in this study has the characteristics of short time and dense data points. This difference allows us to more accurately discover the differences between the time series of different detection conditions. association. Therefore, we believe that the time series similarity measure method can achieve the accurate classification of peroxide explosives based on fluorescence detection time series data.

In this study, we designed a portable fluorescent photoelectric detection system for peroxide explosives using homemade fluorescent probes and used a fusion calculation method combining DDTW and Spearman correlation to analyze and process TATP, H_2_O_2_ and common organic compounds.

## 2. Materials and Methods

In this section, the development of the fluorescent probe and fluorescent photodetection system is discussed, including its design, device selection, fluorescence spectroscopy and fluorescence imaging studies. The methods for field testing this system as well as the following data processing and analysis procedures are also discussed.

### 2.1. Fluorescent Probe

Fluorescent probes were mixed with C6NIB solution, tetrabutylammonium hydroxide (TBAH) solution and absolute ethanol to dilute them to 2 mmol/L as the fluorescent material [29,30]. An aluminum foil-based silica gel plate was used as the substrate, and the fluorescent material was injected into the substrate with a pipette gun and allowed to naturally stand for 5 min. Among them, C6NIB is a kind of naphthalimide polymer that can react with C6NIO after contact with H_2_O_2_ and can produce strong fluorescence effects when excited by light sources of different wavelengths. TBAH can provide an alkaline environment for the reaction and promote the occurrence of the fluorescence effect. Substrate selection explains the fact that manufacturers commonly use peroxide bleaching agents to bleach filter paper, cotton and gauze, which will cause the oxidative bleaching of fluorescent materials. For adsorption and stability, an aluminum foil-based silica gel plate was selected for the substrate.

**Figure 1:**
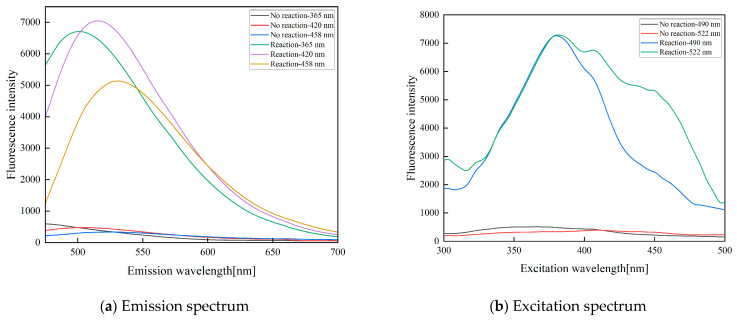
Excitation spectrum and emission spectrum analysis of fluorescent probes, where figure (**a**) shows the emission spectrum under 365 nm, 420 nm and 458 nm excitation light wavelengths, and figure (**b**) shows the excitation spectrum for emission wavelengths of 490 nm and 522 nm.

In accordance with the synthesis mechanism of the fluorescent materials, three excitation wavelengths of 365 nm, 420 nm, and 458 nm were selected, and a Shimadzu RF-6000 spectrometer was used to test the emission and excitation spectra of the fluorescent probes before and after H_2_O_2_ detection. The results of the fluorescence emission spectrum are shown in Figure 1a. According to the analysis, the emission peak under excitation at 365 nm is at 490 nm, and the emission peaks under excitation at 420 nm and 458 nm are at approximately 522 nm. Then, the excitation spectrum was analyzed for the emission wavelengths of 490 nm and 522 nm, and the emission spectra are shown by the black line (before detection) and red line (15 s after detection) in Figure 1b. Excitation wavelengths of 365 nm, 420 nm and 458 nm can be used to achieve more sensitive H_2_O_2_ detection; the light intensity after detection basically increases by more than 6 times, and the fluorescence significantly changes.

**Figure 2:**
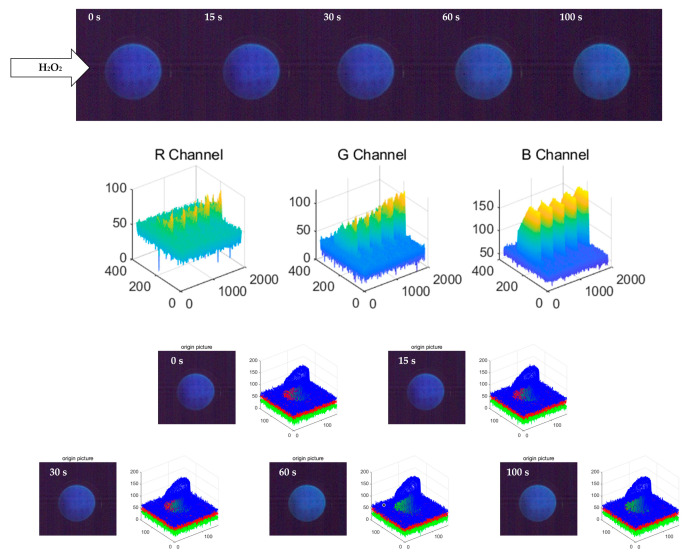
Fluorescence images of fluorescent probes captured by a high-speed camera at 0 s, 15 s, 30 s, 60 s and 100 s, as well as the corresponding RGB images after MATLAB processing. The R channel, G channel and B channel are grayscale images of the red, green and blue components of the fluorescence image, with values ranging from 0 to 255. The RGB images corresponding to 0 s, 15 s, 30 s, 60 s and 100 s are the superposition of the three components of red, green and blue of the fluorescence image.

To explore the changes in fluorescence images during the detection process, this study used a high-speed camera (with a sampling rate of 100 Hz) in the detection of 30% H_2_O_2_ solution to collect continuous images of the fluorescence detection of fluorescent probes and selected the images at 0 s, 15 s, 30 s, 60 s and 100 s, as shown in Figure 2, which were processed via MATLAB to obtain the RGB images at each moment. The comparison shows that the G channel of the fluorescence image grows the fastest, the R channel significantly grows and the B channel is basically unchanged, reflecting the macroscopic performance corresponding to the complete conversion of the fluorescent probe detection group from C6NIB into C6NIO. The change in the G channel can be observed with the naked eye at 60 s, indicating that the fluorescent probe can detect peroxides, which requires a change time of at least 30–60 s, and the needed detection time is longer. However, considering the performance of the high-speed camera itself, as well as the focal length, white balance and other adjustment parameters set when using the acquisition software, it is greatly affected by the experimental environment, white balance and natural light interference, its detection time is long and the response is slow.

To solve the problem of poor stability of the fluorescence image detection method, we focused on the optical signal during the detection process of the fluorescent probe and used a high-sensitivity, low-noise photodetector to collect and perform photoelectric conversion and amplification, which can achieve a fluorescence photoelectric signal gain of ten thousand, thereby improving detection sensitivity and detection stability.

### 2.2. System Design

The design of the fluorescent photoelectric detection system mainly includes having control of the fluorescent reaction flow field of the gas-phase material, the design of the precise optical path structure, driving the high-stability modulated light source, the detection of weak fluorescent signals, the design of a multichannel isolated power supply and the integration of the system master control.

**Figure 3:**
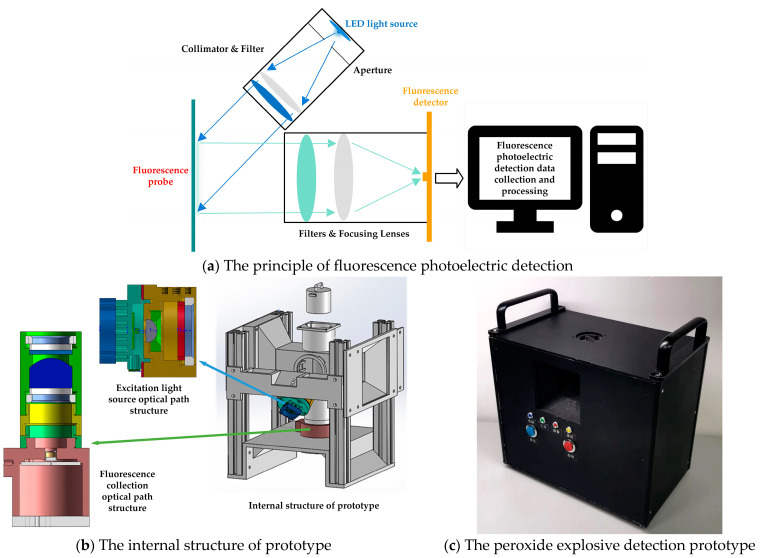
Overall design of the fluorescent photoelectric detection system, including the principle of fluorescence photoelectric detection (**a**), the excitation light source optical path structure, the fluorescence collection optical path structure and the internal structure of the prototype (**b**), and the physical picture of the peroxide explosive detection prototype (**c**).

The overall design of the system is shown in Figure 3. It adopts photoelectric detection technology and takes the photoelectric effect as the physical basis. It mainly includes an optical conversion module, a photoelectric conversion module and a core control circuit module. The electrical signal changes for information transmission and processing are collected via the input circuit, passed through an amplification filter and other detection circuits and input to a microprocessor for calculation and processing through analog-to-digital conversion. The main equipment used in the system is shown in Table 1.

According to the spectral analysis results of the fluorescent probes, combined with the actual application situation, an LED excitation light source with a wavelength of 420 nm is selected, the half width is 10 nm, the power is 1–3 W, the voltage is 3–4.2 V, and the light-emitting surface is approximately 1 mm × 1 mm. The angle is 60°, with optical components such as a bandpass filter, a high-pass filter and various K9 optical lenses employed to design a precise optical path structure, a modulating circuit, and a high-stability modulating light source. A 470 nm high-pass filter is selected, along with a 10,000-fold gain photodetector. The receiving optical signal area is 5 mm × 5 mm, the noise is extremely low, and the detection threshold is large. The fluorescence generated via the excitation of the fluorescent probe can be photoelectrically converted, and the signal can be collected.

A collimating lens and a light source bandpass filter are used in the precise optical path to form a single-wavelength parallel beam. The light source is reflected after being irradiated on the surface of the fluorescent probe. It is concentrated at one point through the high-pass filter and double-cemented lens and is received by the photodetector. The excitation light source is placed on the left side of the experimental table, and the back of the light source is close to a heat sink for heat dissipation. The probe holder is placed 14 cm from the light source at an angle of 90° to the light path of the light source (45° in the fluorescent photoelectric detection system, which has the same effect and is easy to integrate). The fluorescent probe is fixed on the probe holder. The diameter of the parallel light spot obtained from the light source is approximately 10 mm, and after setting the distances between the fluorescent probe and the focusing mirror and between the focusing mirror and the photodetector to 100 mm and 40 mm, respectively, most of the reaction fluorescence can be collected. After the photodetector is connected to the circuit for signal amplification, filtering, and AD conversion, the digital signal data are uploaded to the host computer through the core control circuit for further analysis.

The core control circuit mainly includes photoelectric signal amplification, filtering, modulation, demodulation, AD/DA conversion modules, a microcomputer, an interface and a control module. The signal acquisition and control module uses STM32F4 series processors to realize the control of the working status and signal transmission of each module of the system. The main control power supply is designed as a multichannel isolated power supply, which can provide appropriate voltages for each module and is controlled via the core control module. Finally, each module is precisely integrated through structural design to realize the design of a portable detection system for peroxide explosives.

### 2.3. Data Analysis

In this section, first, according to the sample identifier of the fluorescent photoelectric detection system, the original hexadecimal data are calculated as the corresponding photoelectric voltage values, which can reflect the intensity of the fluorescence generated via the excitation of the fluorescent probe. Using the *DDTW* and Spearman correlation coefficient, the *DDTW* algorithm is used as the main analysis method for similarity measurement, and the fusion calculation with the *Spearman* correlation coefficient improves the ability to analyze the shape similarity and change trend of time series. The calculation formula is

(1) $$DDTW-Spearman=DDTW\times (1-r_{s}),$$

where *r*_s_ is the *Spearman* correlation coefficient and *DDTW* is the *DDTW* distance.

### 2.4. DDTW Algorithm

*DDTW* uses the first-order derivative of the time series instead of the Euclidean distance calculation method used in the classic dynamic time warping (DTW) algorithm, which can effectively solve the problem of singularities encountered with the DTW algorithm, thereby achieving a more accurate time series similarity measurement.

*DDTW* calculates the best regularized path between two time series to align them and obtain the minimum distance after time series regularization. Given the two time series *X* (of length *m*) and *Y* (of length *n*), the regularized matrix, *D*, with dimensions (*m* × *n*) is created as

(2) $$D=\left[\begin{array}{cccc} d(x_{1},y_{1}) & d(x_{1},y_{2}) & \cdots & d(x_{1},y_{n})\\ d(x_{2},y_{1}) & d(x_{2},y_{2}) & \cdots & d(x_{2},y_{n})\\ \vdots & \vdots & \ddots & \vdots \\ d(x_{m},y_{1}) & d(x_{m},y_{2}) & \cdots & d(x_{m},y_{n}) \end{array}\right],$$

where *d*(*x*_i_, *y*_j_) (of which *x*_i_ = (*x*_1_, *x*_2_, …, *x*_m_), *y*_i_ = (*y*_1_, *y*_2_, …, *y*_n_)) represents the dissimilarity measure between *x*_i_ and *y*_j_. The DTW algorithm usually uses the Euclidean distance to calculate the time series similarity after normalization, whereas the *DDTW* algorithm calculates the distance based on the square of the derivative difference, which further improves the robustness of the algorithm.

Taking time series *X* as an example, the *DDTW* algorithm uses the average value of the slopes of the straight lines of the left and right neighbors of a data point as the derivative of the data point, which can increase the generalization of *DDTW*. Additionally, the derivatives of the start and end data points are replaced by the derivatives of the second and penultimate data points, respectively. The derivative calculation formula is

(3) $$\left\{ \begin{array}{l} D_{x}[i]=\frac{\left(x_{i}-x_{i-1})+((x_{i+1}-x_{i-1})/2\right)}{2};1<i<m\\ D_{x}[0]=D_{x}[1]\\ D_{x}[m]=D_{x}[m-1] \end{array}\right.$$

The warping path of the *DDTW* algorithm is a set of continuous matrix elements of the dissimilarity measure between the time series in the regularized matrix, *D*, defined as

(4) $$W=w_{1},w_{2},\cdots ,w_{k},\cdots ,w_{K},$$

which has the following properties:

(1) Boundedness. max(m,n)≤K<m+n−1
(2) Boundary conditions. $w_{1}=(1,1)$, and $w_{k}=(n,m)$.
(3) Continuity. Given $w_{k}=(a,b)$ and $w_{k-1}=(a^{\prime },b^{\prime })$, $a-a^{\prime }\leq 1$ and $b-b^{\prime }\leq 1$ are satisfied.
(4) Monotonicity. Given $w_{k}=(a,b)$ and $w_{k-1}=(a^{\prime },b^{\prime })$, $a-a^{\prime }=0\cap b-b^{\prime }=0$ does not exist.

Finally, according to the above properties, the *DDTW* distance is defined as

(5) DDTW(i,j)=d(i,j)+min[d(i−1,j),d(i,j−1),d(i−1,j−1)]

By calculating the minimum cumulative distance in the global scope, the *DDTW* distance and the shortest warping path are finally obtained, that is, the optimal match between time series *X* and *Y*.

The *DDTW* algorithm used in this study takes the Sakoe–Chiba band as a constraint, keeps the other constraints above unchanged and constrains the time series data image under a fixed coordinate axis to a space range of 4000 data points in length, reducing warping. The calculation range of the path greatly improves the calculation efficiency of the algorithm.

### 2.5. Spearman Correlation Coefficient

The Spearman correlation coefficient is defined as the Pearson correlation coefficient between rank variables (Pearson correlation). For a sample with a sample size of n, after transforming n original data, *X* and *Y*, into grade variables, *R*(*X*) and *R*(*Y*), the Spearman correlation coefficient, *r*_s_, is

(6) $$r_{s}=\rho_{R(X),R(Y)}=\frac{\mathrm{cov}(R(X),R(Y))}{\sigma_{R(X)}\sigma_{R(Y)}},$$

where $\rho_{R(X),R(Y)}$ is the Pearson correlation coefficient calculated using rank variables, cov(R(X),R(Y)) is the covariance of the rank variables, and $\sigma_{R(X)}$ and $\sigma_{R(Y)}$ are the standard deviations of the rank variables.

The Pearson correlation coefficient is used to measure the degree of linear correlation between the variables of the two sets, *X* and *Y*, and is defined as the product of the covariance of the two variables divided by their standard deviations, that is,

(7) $$\rho_{X,Y}=\frac{\mathrm{cov}(X,Y)}{\sigma_{X}\sigma_{Y}}=\frac{E[(X-\mu_{X})(Y-\mu_{Y})]}{\sigma_{X}\sigma_{Y}}.$$

For samples with a sample size of *n*, the Pearson correlation coefficient is also commonly expressed as

(8) $$r=\frac{\sum_{i=1}^{n}\left(X_{i}-\bar{X})(Y_{i}-\bar{Y}\right)}{\sqrt{\sum_{i=1}^{n}{\left(X_{i}-\bar{X}\right)}^{2}}\sqrt{\sum_{i=1}^{n}{\left(Y_{i}-\bar{Y}\right)}^{2}}}$$

In this study, the piecewise Spearman correlation coefficient is used to divide each of the two groups of time series data into 20 segments according to the time length, and the correlation calculation is performed for each segment. Finally, the 20 segment correlation coefficients are weighted and averaged to obtain the final Spearman correlation coefficient.

### 2.6. Computational Environment

The computational environment for the DTW algorithm, *DDTW* algorithm and Spearman correlation is in NVIDIA RTX 3060. The development environment of the algorithm was Python 3.11.5 using PyTorch 2.0 with CUDA 11.8.

## 3. Results

In this section, the stability test of the fluorescent photoelectric detection system and the experimental results of TATP, equivalent H_2_O_2_ solution and organic compound detection are presented. The DTW–Spearman distance and DDTW–Spearman distance results are calculated using the DTW algorithm, DDTW algorithm and Spearman correlation coefficient fusion, which are used to analyze and improve the sensitivity of TATP detection.

### 3.1. Stability

**Figure 4:**
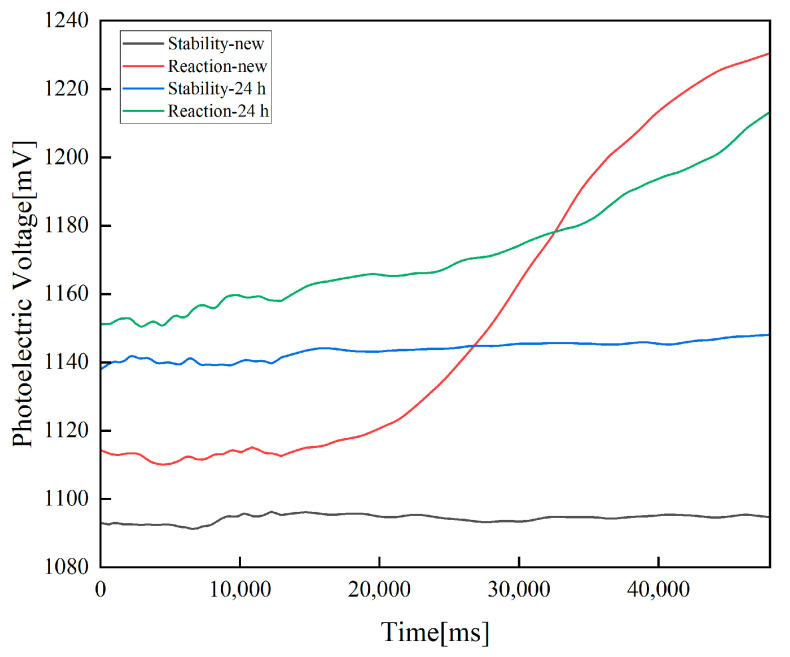
Results of detection experiments with freshly prepared fluorescent probes and probes stored for 24 h. The x-axis is the time, with a unit of milliseconds (ms), and the y-axis is the photoelectric voltage reflecting the fluorescence intensity, with a unit of mV. The system signal acquisition method includes the detection baseline calculation stage before 12,950 ms and the detection stage after 12,950 ms. The baseline is the weighted average of 8000 voltage data from 2000 ms to 9999 ms.

Using the newly prepared fluorescent probe and the fluorescent probe sealed and stored in a vacuum/oxygen-free, light-proof, constant-temperature environment of 20 °C for 24 h, an empty detection experiment and an inhalation detection experiment using 3% H_2_O_2_ solution were carried out. The experimental results are shown in Figure 4. Via calculation, the empty detection baseline of the newly prepared fluorescent probe is 1092.17948 mV, and the maximum value is 1094.59609 mV. The baseline of H_2_O_2_ detection is 1111.62033 mV, and the detection curve monotonically rises, with a maximum value of 1230.72004 mV. The empty detection baseline of the fluorescent probe stored for 24 h is 1140.31543 mV, and the maximum value is 1148.16445 mV. The baseline of H_2_O_2_ detection is 1153.16334 mV, and the detection curve monotonically rises, with a maximum value of 1213.78298 mV.

Within 35 s of the detection process, the newly prepared fluorescent probe has excellent stability, the change in the empty detection does not exceed 3 mV and the probe is extremely sensitive to H_2_O_2_. The change trend is obvious, and the growth value exceeds 120 mV. After being stored for 24 h, the fluorescent probe still has good stability, the change in the empty detection is not more than 10 mV, the probe is sensitive to H_2_O_2_ and the growth value exceeds 60 mV. In the follow-up studies of this study, newly prepared fluorescent probes were used to maintain the most stable and sensitive state of the fluorescent photoelectric detection system.

In addition, it can be seen that the initial voltage value of each detection in Figure 4 is different, causing the detection baseline of each detection to shift. The main reasons for this situation are as follows:

1. The prototype will be powered on and off again after each test and there will be slight deviations in the working status, which is a mechanical error. This is also the main reason why the detection baseline shifts when using the same fluorescent probe for empty detection and H_2_O_2_ detection.
2. When using different fluorescent probes, since the preparation process of fluorescent probes has not yet been industrialized, there are certain deviations in artificial preparation. For example, the concentration distribution of fluorescent materials is not uniform enough, resulting in slight differences in fluorescence signal collection after the replacement of fluorescent probes.

To solve the impact of the baseline shift, we have adopted three methods to improve the stability of fluorescent probes on the basis of continuously optimizing the hardware design, which are as follows:

1. After fixing and replacing the fluorescent probe for the first time, a full-process empty detection experiment including the baseline calculation phase and the detection phase, such as stability-new and stability-24 h detection in Figure 4, is conducted to verify the stability of the fluorescent probe. Stability without the influence of the test object is determined.
2. In a single detection, a 20 s empty detection is first performed to calculate the baseline of the detection.
3. In a single detection, for both the baseline calculation phase and the detection phase, sampling starts after 5 s, effectively reducing the impact of high noise when the prototype is started.

Finally, in the prototype design stage, since our detection judgment is based on the change value and trend of the detection curve, the baseline offset problem caused by the influence of the initial voltage value can be completely solved using the above method.

### 3.2. Specificity

**Figure 5:**
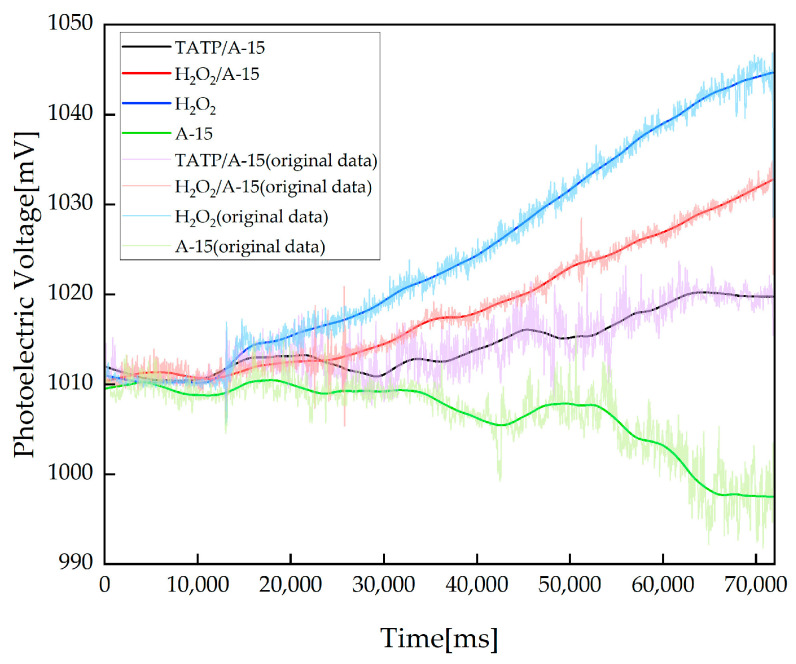
Results for the detection of the TATP/A-15 solution, A-15 solution, equivalent H_2_O_2_/A-15 solution and H_2_O_2_ solution. The detection baseline calculation stage and the detection stage are the same as those in the stability experiments.

Since TATP is not easy to obtain, this study adopted the swab sampling method with a sampling volume of 1 μL and carried out detection experiments with 0.01 mg/mL of TATP/A-15 (hereinafter referred to as TATP/A-15) solution, 0.0046 mg/mL of H_2_O_2_/A-15 (hereinafter referred to as H_2_O_2_/A-15) solution, and 0.0046 mg/mL of H_2_O_2_ (hereinafter referred to as H_2_O_2_) solution and A-15 solution. The experimental results are shown in Figure 5. The concentration of H_2_O_2_ produced via the complete decomposition of 0.01 mg/mL of TATP solution is 0.0046 mg/mL, and the equivalent H_2_O_2_ solution is prepared by diluting 30% H_2_O_2_ solution 65,210 times. The H_2_O_2_ concentration reaches 1.314 ppb, and the equivalent TATP solution concentration is lower than 1 ppm and close to 1 ppb.

The detection curves for the TATP/A-15 solution, H_2_O_2_/A-15 solution, and H_2_O_2_ and A-15 solution have different trends. Nonmonotonic changes may exist by chance. Therefore, the implicit correlation between the detection of the TATP solution and the detection of the equivalent H_2_O_2_ solution must be further analyzed.

**Figure 6:**
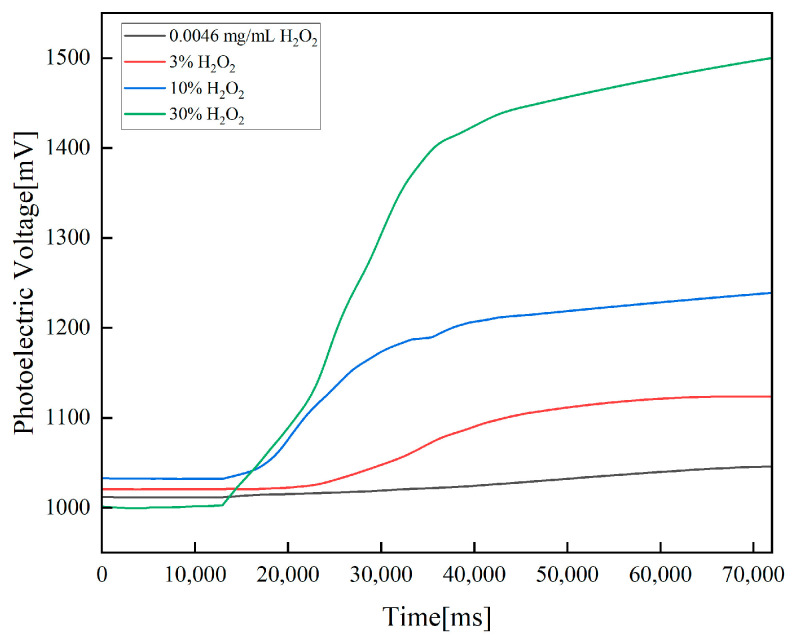
Results for the detection of 0.0046 mg/mL of H_2_O_2_, 3% H_2_O_2_, 10% H_2_O_2_ and 30% H_2_O_2_. The detection baseline calculation stage and the detection stage are the same as those in the stability experiments.

Then, detection experiments were conducted using 0.0046 mg/mL of H_2_O_2_ solution, 3% H_2_O_2_ solution, 10% H_2_O_2_ solution and 30% H_2_O_2_ solution. The experimental results are shown in Figure 6. Via calculation, the detection baseline of 0.0046 mg/mL of H_2_O_2_ is 1011.59240 mV and the detection curve rises monotonically, with the maximum value being 1045.89319 mV. The detection baseline of 3% H_2_O_2_ is 1020.62446 mV, the detection curve rises monotonically and the maximum value is 1123.72985 mV. The detection baseline of 10% H_2_O_2_ is 1032.39993 mV, and the detection curve rises monotonically, with a maximum value of 1238.76456 mV. The detection baseline of 30% H_2_O_2_ is 1000.3367 mV, the detection curve rises monotonically and the maximum value is 1499.70406 mV. The growth value of 0.0046 mg/mL H_2_O_2_, 3% H_2_O_2_, 10% H_2_O_2_ and 30% H_2_O_2_ are 34.38550 mV, 103.19401 mV, 206.70932 mV and 500.21114 mV, respectively.

**Figure 7:**
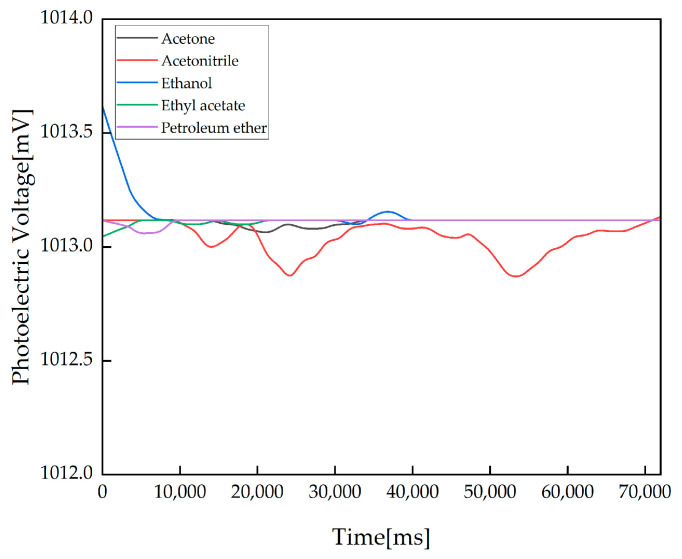
Results for the detection of acetone, acetonitrile, ethanol, ethyl acetate and petroleum ether. The detection baseline calculation stage and the detection stage are the same as those in the stability experiments.

At the same time, acetone, acetonitrile, ethanol, ethyl acetate and petroleum ether were used to measure common organic compounds. The experimental results are shown in Figure 7. The detection curves for the five organic compounds, including as acetone, do not change by more than 1 mV, indicating that the detection system is not interfered with by the five organic compounds acetone, acetonitrile, ethanol, ethyl acetate, and petroleum ether.

### 3.3. Sensitivity

**Figure 8:**
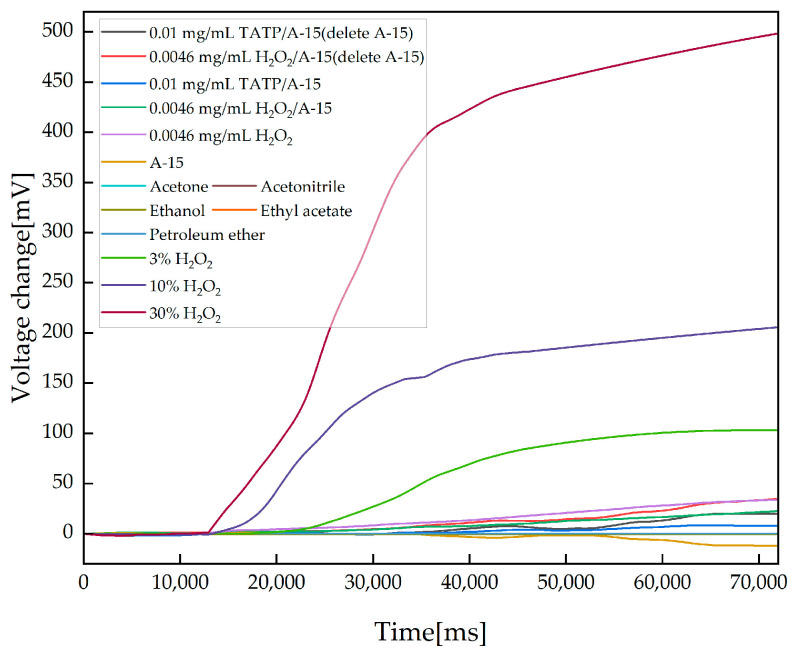
Results obtained after processing the data from the detection experiments for TATP/A-15 (A-15 deleted), TATP/A-15, A-15, equivalent H_2_O_2_/A-15 (A-15 deleted), H_2_O_2_/A-15, H_2_O_2_, acetone, acetonitrile, ethanol, ethyl acetate, petroleum ether, 3% H_2_O_2_, 10% H_2_O_2_ and 30% H_2_O_2_.

The A-15 detection data were subtracted from the TATP/A-15 and H_2_O_2_/A-15 detection data to obtain the data for TATP/A-15 (A-15 deleted) and H_2_O_2_/A-15 (A-15 deleted). The initial value of the data was uniformly reset to zero to obtain TATP/A-15 (A-15 deleted), H_2_O_2_/A-15 (A-15 deleted), TATP/A-15 and H_2_O_2_/A-15 data. The detection data of H_2_O_2_, A-15, acetone, acetonitrile, ethanol, ethyl acetate, petroleum ether, 3% H_2_O_2_, 10% H_2_O_2_ and 30% H_2_O_2_ are shown in Figure 8.

**Figure 9:**
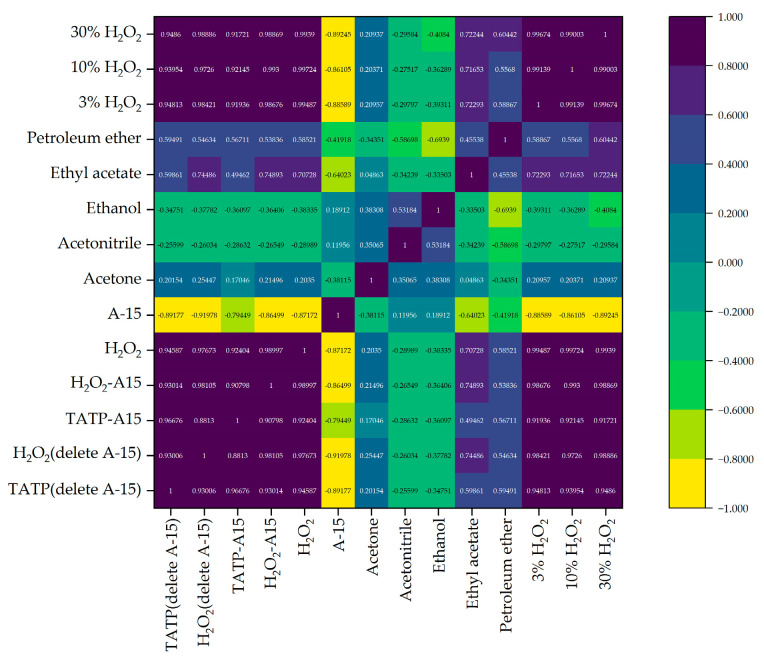
Spearman correlation coefficient calculated from the detection data of TATP/A-15 (A-15 deleted), H_2_O_2_/A-15 (A-15 deleted), TATP/A-15, H_2_O_2_/A-15, H_2_O_2_, A-15, acetone, acetonitrile, ethanol, ethyl acetate, petroleum ether, 3% H_2_O_2_, 10% H_2_O_2_ and 30% H_2_O_2_.

The Spearman correlation coefficient between the above data was calculated. The results are shown in Figure 9. The detection data of TATP/A-15 (A-15 deleted), TATP/A-15, H_2_O_2_/A-15 (A-15 deleted), H_2_O_2_/A-15, H_2_O_2_, 3% H_2_O_2_, 10% H_2_O_2_ and 30% H_2_O_2_ are shown in Figure 9. The correlation coefficients are all greater than 0.85, which indicates an extremely strong correlation. Moreover, the correlation coefficients between them and A-15, acetone, acetonitrile, ethanol, ethyl acetate and petroleum ether range from −0.9 to 0.75, which cannot be clearly distinguished.

**Figure 10:**
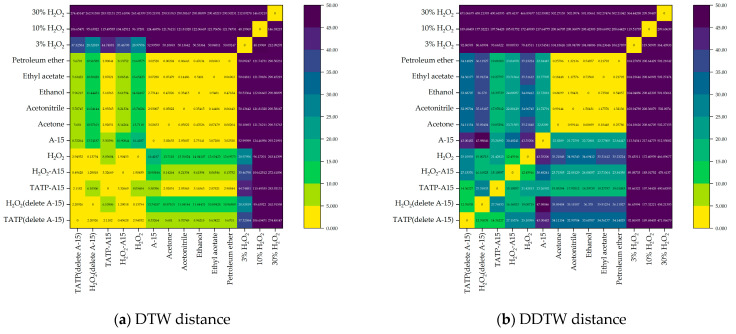
DTW distance (**a**) and DDTW distance (**b**) calculated from the detection data of TATP/A-15 (A-15 deleted), H_2_O_2_/A-15 (A-15 deleted), TATP/A-15, H_2_O_2_/A-15, H_2_O_2_, A-15, acetone, acetonitrile, ethanol, ethyl acetate, petroleum ether, 3% H_2_O_2_, 10% H_2_O_2_ and 30% H_2_O_2_.

The DTW distance and DDTW distance between the above data were calculated. The results are shown in Figure 10. In Figure 10a, the DTW distances between the detection data of 3% H_2_O_2_, 10% H_2_O_2_ and 30% H_2_O_2_ and other detection data are all greater than 25. The DTW distances between the detection data of acetone, acetonitrile, ethanol, ethyl acetate and petroleum ether are all less than 3.5, and there is no obvious relationship between other data. In Figure 10b, the DDTW distances between the detection data of 3% H_2_O_2_, 10% H_2_O_2_ and 30% H_2_O_2_ and other detection data are all greater than 75. The DDTW distances between the detection data of acetone, acetonitrile, ethanol, ethyl acetate and petroleum ether are all less than 2, and there is no obvious relationship between other data.

**Figure 11:**
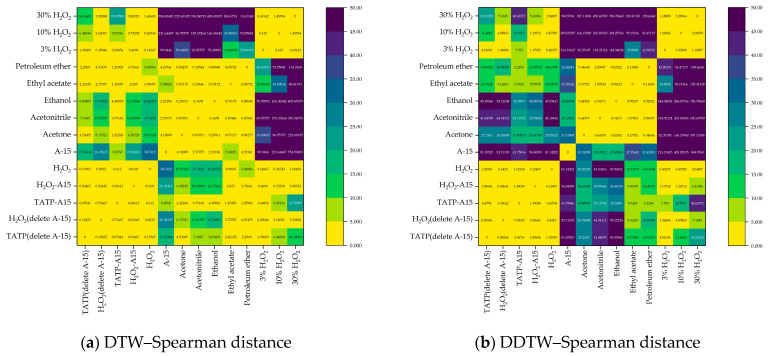
DTW–Spearman distance (**a**) and DDTW–Spearman distance (**b**) calculated via fusion of the DTW distance, DDTW distance and Spearman correlation coefficient.

The DTW–Spearman distance and DDTW–Spearman distance can be obtained by calculating and merging the DTW distance, DDTW distance and the Spearman correlation coefficient, as shown in Figure 11.

In Figure 11a, the DTW–Spearman distance can clearly distinguish the detection data of 3% H_2_O_2_, 10% H_2_O_2_, and 30% H_2_O_2_ from acetone, acetonitrile, ethanol, ethyl acetate and petroleum ether. However, due to the small changes in the detection data of TATP/A-15 (A-15 deleted), TATP/A-15, H_2_O_2_/A-15 (A-15 deleted), H_2_O_2_/A-15 and H_2_O_2_, they are different from the DTW–Spearman distances between acetone, acetonitrile, ethanol, ethyl acetate and petroleum ether, and 3% H_2_O_2_, 10% H_2_O_2_ and 30% H_2_O_2_, so they cannot be used in similarity measurement. The detection data of analytes containing H_2_O_2_ are classified into one category and cannot be used as a basis for detection.

In Figure 11b, the DDTW–Spearman distance can clearly distinguish the detection data of 3% H_2_O_2_, 10% H_2_O_2_ and 30% H_2_O_2_ from acetone, acetonitrile, ethanol, ethyl acetate and petroleum ether. The DDTW–Spearman distances between the detection data of TATP/A-15 (A-15 deleted), TATP/A-15, H_2_O_2_/A-15 (A-15 deleted), H_2_O_2_/A-15 and H_2_O_2_ are all less than 3.5. The DDTW–Spearman distances between the detection data of acetone, acetonitrile, ethanol, ethyl acetate and petroleum ether are all less than 2.5. The DDTW–Spearman distances between the detection data of 3% H_2_O_2_, 10% H_2_O_2_ and 30% H_2_O_2_ are all less than 2.5. Moreover, the DDTW–Spearman distance between the detection data of H_2_O_2_-containing test substances other than 30% H_2_O_2_ can be clearly distinguished from those for acetone, acetonitrile, ethanol, ethyl acetate and petroleum ether, enabling the effective detection of TATP and H_2_O_2_.

## 4. Discussion

Among the explosive detection technologies, infrared spectroscopy, Raman spectroscopy, electrochemistry, ion mobility spectroscopy, mass spectrometry and liquid chromatography all use trace explosives enriched for corresponding spectral analysis, and chemical colorimetry uses color image analysis methods. Fluorescence detection technology uses the fluorescence generated via the excitation of fluorescent probes, and methods such as spectral analysis, fluorescence image analysis and fluorescence photoelectric detection can be used. Among them, fluorescence spectral analysis and fluorescence image analysis require a fluorescence spectrophotometer or a high-precision optical camera after trace explosives are enriched. Fluorescence photoelectric detection can use optical path focusing to detect explosives in real time, the cost required is lower than that of fluorescence spectrophotometers and optical cameras, and the timeliness of detection can be ensured.

The fluorescent photoelectric detection system designed in this study uses a fusion calculation method combining the DDTW algorithm and Spearman correlation coefficient to analyze the changes in fluorescent photoelectric signals to realize the efficient and sensitive detection of peroxide explosives. The fluorescent photoelectric signal is a set of data arranged in order according to a certain time interval, which is a typical time series. Time series similarity measurement is the basis for similarity retrieval, unsupervised clustering, classification and other analyses of time series. By analyzing the time series similarity, shape similarity and change similarity of time series, the similarity relationship between two time series can be effectively measured. Among them, time series similarity means that the increase and decrease change patterns of time series points are the same, that is, they increase or decrease at the same time point, and the Spearman correlation coefficient can effectively reflect this similarity. Shape similarity refers to having common shapes in time series, usually including common trend shapes occurring at different time points or subpatterns in the data that are the same independent of the time point. Change similarity refers to the same change law of a time series from one time point to the next time point. The DDTW algorithm realizes the measurement of time series shape similarity and change similarity via the replacement of the Euclidean distance with derivatives. However, few studies have utilized time series analysis methods for explosive detection and identification. Taking common explosive detection technology as an example, Raman spectroscopy technology is based on the Raman scattering effect and uses a laser to excite the object to be measured to obtain a Raman spectrum with specific vibration characteristics of the molecule to achieve the qualitative and quantitative analysis of explosives. IMS technology ionizes the substance to be measured into ions, and obtains migration spectra based on the drift time of different substances through a uniform weak electric field at a fixed distance. Colorimetric technology uses colorimetric probes to detect explosives based on the color change produced after the probe reacts with the analyte. MS technology ionizes the analyte and then passes it through a mass analyzer according to the mass-to-charge ratio (mass–charge ratio) of the ions to obtain a mass spectrum for analysis. Among the data for these explosive detection technologies, there are no time series data for Raman spectroscopy, mobility spectrometry and mass spectrometry. Colorimetry analyzes color changes. After using an extremely high-performance image acquisition module, time series images can be obtained. However, currently limited by image acquisition modules such as CCD cameras, the time series similarity measurement method cannot yet be applied. The reason why there are so few published articles may be that such methods cannot be applied in most explosive detection techniques.

In this study, by setting the appropriate DDTW–Spearman distance classification standard, the classification of different test objects is achieved. For example, the DDTW–Spearman distance between two sets of data is 3.5 for classification. When the distance is less than 3.5, it indicates that the two sets of data are in the same category, and vice versa. Thus, it can be classified into low-concentration peroxide detection categories such as TATP/A-15 (A-15 deleted), TATP/A-15, H_2_O_2_/A-15 (A-15 deleted), H_2_O_2_/A-15 and H_2_O_2_, and organic matter detection categories for acetone, acetonitrile, ethanol, ethyl acetate and petroleum ether, catalyst detection categories for A-15, and high-concentration peroxide detection categories for 3% H_2_O_2_, 10% H_2_O_2_ and 30% H_2_O_2_. At the same time, the DDTW–Spearman distance between the detection data of 0.0046 mg/mL H_2_O_2_, 3% H_2_O_2_, 10% H_2_O_2_ and 30% H_2_O_2_ does not exceed 3, and these detection data are consistent with those of A-15, acetone, acetonitrile, ethanol and ethyl acetate. The DDTW–Spearman distances between the ester and petroleum ether detection data are both greater than 10. The research results show that the detection limit of the fluorescence photoelectric detection system designed in this study is <10 ppb for 0.0046 mg/mL of H_2_O_2_ and 0.01 mg/mL of TATP. The time series similarity measurement method studied can achieve the effective classification of peroxides and has the ability to use H_2_O_2_ detection to replace TATP for effective detection.

However, there are some limitations to the retrospective design of this study. In the result, the baseline drift problem caused by the change of the initial value indicates that the prototype and fluorescent probe can be further optimized. The industrial production of fluorescent probes, standardized design of prototypes and big data analysis of artificial intelligence models can be used to establish automatic calibration with an initial status and a functional explosives analysis model. At the same time, the fluorescent probe used in this study has problems such as its short life. The fluorescent probe coating method can be improved (such as via spin coating) to make the fluorescent material more evenly distributed, and appropriate antioxidants can be added to improve the stability of the fluorescent probe. The attenuation model of fluorescent probes affected by environmental influences such as light and heating can be established. The time series similarity measurement method proposed in this study relies on the global information of the data, and can use artificial intelligence methods to establish a multidimensional time series big data real-time analysis model by setting up many experiments with different conditions. In addition, due to the lack of stability of most peroxide explosives (such as TATP and HMTD), they are extremely difficult to obtain. Compared with machine learning and deep learning, time series similarity measurement methods do not require a large number of samples. After experimentally verifying the similar metric relationship between peroxide explosives such as TATP and HMTD and H_2_O_2_ detection, the equivalent H_2_O_2_ detection experiment can be performed using the DDTW–Spearman distance calculation method combined with the artificial intelligence model in this study. This enables the detection and identification of various peroxide explosives. At the same time, it can greatly reduce the calculation amount of artificial intelligence models and improve the versatility and accuracy of time series similarity measurement methods.

## 5. Conclusions

Fluorescence detection technology is widely used in the detection and identification of trace peroxide explosives. At the same time, the time series similarity measurement method has also been widely developed in the fields of data mining and cluster analysis, but it has not been effectively applied in research on explosive detection technology.

In this study, we designed a fluorescent photoelectric detection system for the detection of trace peroxide explosives, developed a prototype and provided a classification method based on fusion calculation of the DDTW algorithm and Spearman correlation coefficient, revealing the similarity measure between the detection of H_2_O_2_, A-15, acetone, acetonitrile, ethanol, ethyl acetate and petroleum ether. However, this method has limitations, and more types of peroxide explosives and organic compounds, such as diacetone diperoxide (DADP), HMTD and methane, must be classified to further improve and promote its application in real detection environments.

This study demonstrates the broad application prospects of fluorescence detection technology in the detection of trace peroxide explosives and the possibility of applying time-series analysis methods in the detection of explosives.

## Figures and Tables

**Table 1:** Experimental equipment.

Equipment	Quantity	Parameters
optical bench	1	600 mm × 600 mm × 50 mm
power supply	1	220 V AC
LED light source	3	420 nm
bandpass filter	3	420 ± 25 nm
high-pass filter	1	470 nm
iris diaphragm	1	M4, F2-29
pipette gun	2	0.5–10 μL, 10–100 μL
pipette tips	some	10 μL, 200 μL
detector	1	10,000-fold gain

## Data Availability

The data presented in this study are available on request from the corresponding author. The data are not publicly available due to privacy constraints.
